# Bearing Characteristics of Screw-Groove Piles: Model Test and Numerical Analysis

**DOI:** 10.3390/ma17235791

**Published:** 2024-11-26

**Authors:** Huiling Zhao, Yousheng Deng, Ziying Zhuang, Zhigang Yao

**Affiliations:** 1 School of Architecture and Civil Engineering, Xi’an University of Science and Technology, Xi’an 710054, China; dengys10@163.com (Y.D.); zhuangzy100@163.com (Z.Z.); yaozg100@163.com (Z.Y.); 2 Pile-Supported Structures Research & Test Center, Xi’an University of Science and Technology, Xi’an 710054, China

**Keywords:** screw-groove pile, structure optimization, bearing capacity, experimental study, finite element method, characteristics analysis

## Abstract

Screw-groove piles, a new type of precast pile, are economically and environmentally friendly and improve the load-bearing performance of piles through a unique screw-groove structure. To reveal the load-transfer characteristics and bearing mechanism of the screw-groove pile, the axial force, load–settlement curve, skin friction, bearing capacity, and response characteristics of the foundation for piles under vertical loading were analyzed. Furthermore, a parameter analysis of the ultimate bearing capacity and material utilization of screw-groove piles was performed using the finite element method. The results demonstrate that the screw-groove pile had an ultimate bearing capacity 1.85 times higher than that of the circular pile, and its material utilization rate was 2.85 times higher. The screw-groove surface end resistance and pile-tip resistance formed a multipoint vertical bearing mode. It efficiently utilized the soil’s shear strength and mobilized a larger volume of surrounding soil to share the load. The screw-groove structure increased the pile–soil interaction surface, thereby increasing the skin friction resistance of the pile. Additionally, increasing the inner radius of the screw groove boosts the pile’s bearing capacity but may reduce material utilization. An optimal screw-groove spacing balances both factors, while excessive groove thickness lowers material use. The pile shows high sensitivity to soil parameters.

## 1. Introduction

As engineering projects become more ambitious and intricate, the demand for construction piles with enhanced bearing capacity has increased [1]. Pile foundations are primarily constructed using bored or precast piles [2]. However, problems such as difficulty in quality control of bored piles, generation of mud pollution, and consumption of large amounts of resources are particularly prominent, making the use of these piles difficult to align with the principles of sustainable development [3]. Compared with bored piles, precast concrete piles are more aligned with current international green industrial development because of their advantages, such as simple and fast construction, full utilization of material properties, and strong adaptability to geological conditions [4,5,6].

Traditional precast concrete piles consume a significant amount of concrete, and studies have indicated that emissions associated with cement production contribute up to 8% of global carbon emissions [7,8]. Moreover, recent research suggests that the widespread adoption of zero- or low-carbon cement in the coming decades is unlikely [9]. Consequently, many researchers have focused on developing innovative pile–foundation designs, reducing concrete consumption, and increasingly achieving efficient material utilization in research and practical applications [10,11]. Lv et al. [12] conducted experimental research using a model test to study the bearing capacity of X-section concrete piles compared to traditional circular piles. They demonstrated that the skin friction of X-section piles was greater than that of circular piles, with a perimeter approximately 1.46 times that of circular piles. However, the soil arching effect in concave arc sections partially mitigated the skin friction resistance of the X-section piles. Wang et al. [13] and Li et al. [14] analyzed the bearing capacity of Y-shaped piles and plum-blossom piles. The research showed that the ultimate bearing capacity and the lateral surface area of Y-shaped piles were 1.6 times and 1.56 times higher than those of circular piles. The pile end resistance, skin resistance, and settlement of plum-blossom piles were 1.0, 1.38, and 1.12 times higher than those of circular piles. Haataf and Shafaghat [15] found that the bearing capacity of tapered piles is 11% higher than that of circular piles with the same volume, and in dense sands, the optimal pile tapering angle is 1.2°. Abushama et al. [11] compared the environmental impact of concrete circular piles and tapered piles based on a genetic algorithm, and the results showed that the carbon emission of piles can be reduced by more than 58% when the optimal tapering angle is adopted. Based on an on-site test, Zhou et al. [3] showed that the average skin friction of static prebored grouted planted piles was 1.28 times the average skin friction of circular piles, and the construction efficiency was higher for piles of the same diameter. Aamer et al. [16] introduced a blade pile anchor designed to enhance the surface area in contact with the surrounding soil, thereby improving shear resistance during uplift. Their study demonstrated that the uplift capacity of the blade pile anchor increased by approximately 230% to 275% compared to conventional regular piles. Bak et al. [17] proposed the tapered helical pile, integrating the benefits of both tapered and blade pile designs. The research findings indicate that the friction resistance of the pile increases with settlement, and that the material utilization efficiency of the tapered helical pile is 2.5 times greater than that of a regular pile with a similar quantity of material. Chen et al. [18] proposed composite step piles with H-shaped and rectangular sections. The experimental results indicate that these composite piles can reduce pile length, save materials, and exhibit a greater plastic deformation capacity within pile–soil systems.

Innovative optimization of pile foundations mainly enhances load-bearing capacity by increasing the toe or skin friction resistance [19], aiming to maximize the potential load-bearing capacity of both the foundation soil and the pile itself [20,21,22], thereby reducing resource consumption. Based on this, this study proposes a new type of precast screw-groove pile intended to enhance pile capacity by combining pile skin resistance and toe resistance during load transfer, fully utilizing the inherent shear strength of the soil and significantly improving the resource utilization efficiency. The optimized pile foundation design considers material efficiency and offers a new possibility for designing modernized, green-assembled precast piles.

In this study, experimental and numerical analyses were conducted to investigate the bearing mechanisms of screws and circular piles under vertical loads. The differences in the load–settlement, axial force, skin friction resistance, pile–soil displacement, plastic deformation, and stress field distribution between the two types of piles were comparatively analyzed. Furthermore, a parameter analysis of the bearing performance of the proposed screw-groove piles was performed using the finite element method (FEM), investigating the effects of the inner radius of the screw groove, the screw-groove spacing, the thickness of the screw groove, the angle of internal friction, and cohesion. From the perspective of material utilization efficiency, the aim was to provide theoretical reference data for the design and application of such piles. Finally, this study analyzed the influence of various screw-groove geometric parameters on material utilization efficiency, aiming to provide a foundation for the design and application of such piles.

## 2. Materials and Methods

### 2.1. Specimen Setup and Materials

**Figure 1:**
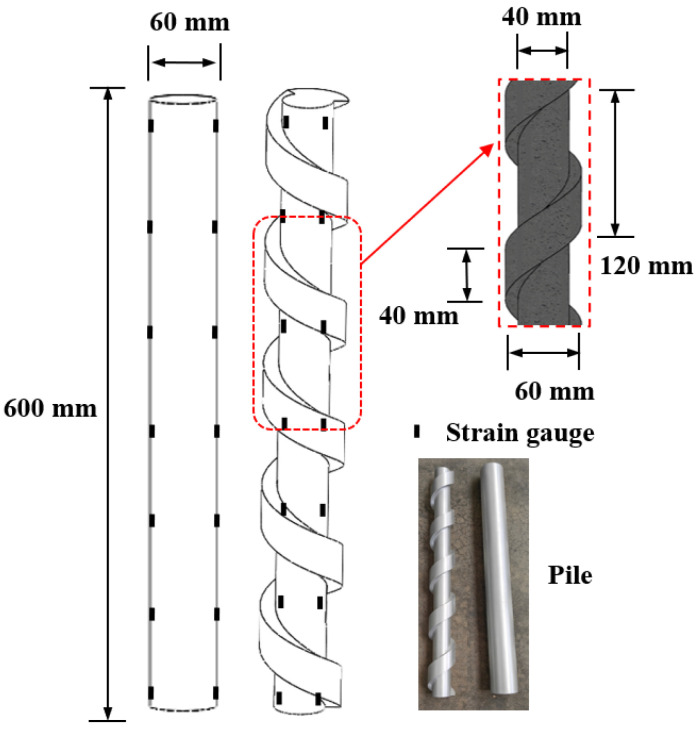
Schematic diagram of model pile.

Tests were conducted using a self-developed multifunctional pile foundation model box with a width and height of 2 m and a length of 4 m. Equal-angle steel was welded to the upper part of the box, two sides of which were built with transparent toughened glass. The test pile was located in the middle of the model box at a distance greater than ten times the pile diameter, and the boundary effect was neglected [23]. To investigate the effect of pile type on the vertical bearing law of piles, screw-groove piles and circular piles were designed. Current model tests for piles are primarily simulated using materials such as aluminum tubes [24], engineering plastics [25], and artificial stone [26]. Aluminum tubes can better simulate concrete in terms of the modulus of elasticity and strength, and the material is easy to process to produce strains that meet test requirements. Therefore, the test screw-groove pile and circular pile were made of aluminum tubes, as shown in Figure 1. The test screw-groove pile length was 600 mm, the inner diameter was 40 mm, the outer diameter was 60 mm, the width of the screw groove was 10 mm, the thickness of the screw groove was 40 mm, the spacing was 120 mm, and the circular pile had a length of 600 mm and a diameter of 60 mm. The surface of the model pile was coated with epoxy resin and mortar to approximate the rough contact between the pile and the soil. In this study, the soil was loess from construction sites in the Lintong District, Xi’an City, which was a fine-grained loess after screening. The density of the loess was 1.81 g/cm^3^, the cohesion was 33.3 kPa, and the friction angle was 23.4°, as determined by performance tests such as the quadruple straight shear test.

### 2.2. Testing Procedures

**Figure 2:**
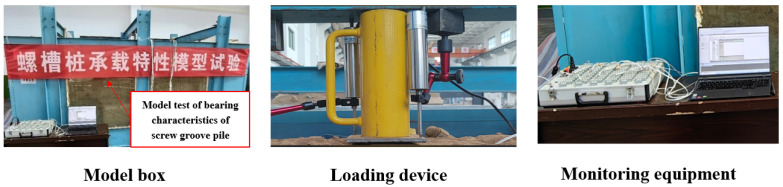
Model test system.

As shown in Figure 2, the test loading device consisted of a static loading system, monitoring equipment, and a model box. A static force was applied to the pile using the loading system consisting of reaction frames, jacks, and loading plates on the upper part of the model box. The monitoring equipment included strain gauges, settlement meters, and data-acquisition devices. A DH5922D stress–strain analyzer obtained from Jingjiang Donghua Co. Ltd. (Taizhou, China) was used for test acquisition, and the maximum sampling frequency of the acquisition device was 50 Hz. This study used a slow-sustaining loading method for graded loading. The loading standard refers to building pile testing technology code JGJ106-2014 [27]. Readings were taken at the 5th, 15th, and 30th min of loading, after which readings were taken once every 30 min. A settlement meter measured the settlement at the top of the pile. When two settlements did not exceed 0.01 mm/30 min, the next level of loading could be performed. Seven uniformly spaced strain gauges were symmetrically pasted on the screw-groove pile and the circular pile to monitor the strains along the buried depth direction under graded loads. Insulating tape protected the strain gauges and collection wires from damage during loading.

### 2.3. Numerical Simulation Models

**Figure 3:**
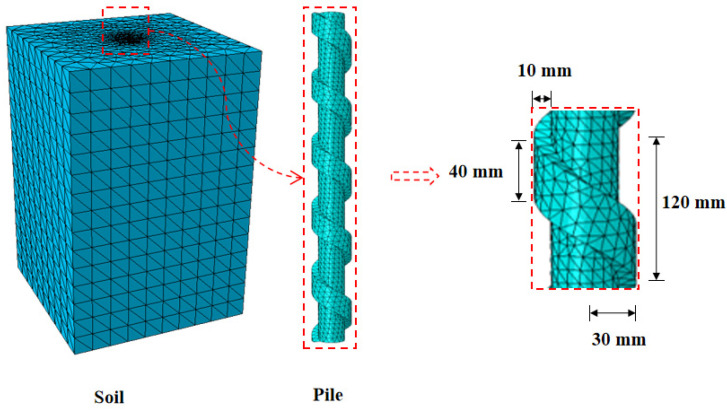
Geometric modelling and meshing of numerical model.

The main objective of the numerical models was to obtain insight into the load-transfer mechanism of screw-groove piles under vertical loads. The FEM provided the effects of loading on the surrounding soil and pressure beneath the screw groove. The FEM software ABAQUS 2020 established a three-dimensional numerical model with the same dimensions as the test. The screw-groove pile has a complex structure. To ensure the integrity of the pile structure, the screw and scanning excision functions of the software were used to establish a three-dimensional geometric model of the screw-groove pile. The mesh was divided using a 10-node modified tetrahedron with an hourglass control. To ensure a uniform mesh size at the pile–soil interface, mesh refinement was applied to the surrounding soil around the screw-groove pile. The model was iteratively refined to balance simulation accuracy and computational efficiency, resulting in 84,001 elements. The numerical model and mesh divisions are shown in Figure 3. The pile was modelled using a linear elastic material. In contrast, the soil was simulated using an elastoplastic constitutive model based on the Mohr–Coulomb yield criterion [28]. The pile–soil interface was defined as a surface-to-surface contact, with tangential behavior enforced using penalty functions and normal behavior set as hard contact. The friction coefficient was determined as the tangent of the interface friction angle, according to Randolph and Worth [29]. The material parameters of the soil and pile are listed in Table 1. In addition, to simulate the actual conditions of the test, the boundary of the bottom surface was assumed to be restricted in the X, Y, and Z directions. The side and symmetry boundaries were fixed in the X and Y directions. The pile was loaded incrementally based on the equilibrium of the ground stresses.

## 3. Results and Discussion

### 3.1. Material Utilization Ratio

**Figure 4:**
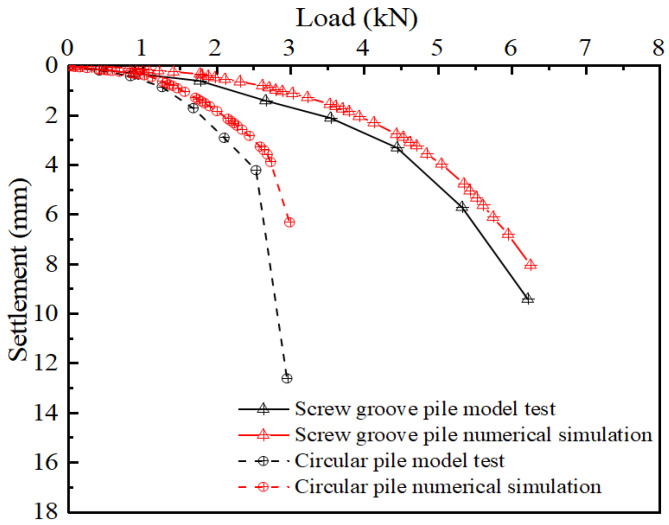
Load–settlement curves of numerical simulations and model tests.

The ultimate bearing capacity of the pile was determined based on the load–settlement curves obtained from the tests, as shown in Figure 4, for both the circular and screw-groove piles. In the early loading stage, the two settlement curves coincided, showing a gentle linear change. At this stage, the pile was less loaded, and the pile type had little influence on the bearing capacity. As the load increased, the settlement of the screw-groove pile gradually became smaller than that of the circular pile, and the occlusion of the screw groove with the soil slowed the settlement of the screw-groove pile. The load–settlement curve of the circular pile showed a steep drop in the later stages of loading, and the ultimate capacity of the pile was 2.54 kN. The screw-groove pile exhibited prominent slow-change characteristics compared with the circular pile; according to building pile testing technology code JGJ106-2014, the slow-change load–settlement curve of the pile should be selected to settle 40 mm for its ultimate bearing capacity. When the top of the pile settlement reached 4 mm (a geometric similarity ratio of 10), the ultimate bearing capacity of the screw-groove pile was 4.71 kN. The ultimate bearing capacity of the screw-groove pile was increased by 1.85 times compared to that of the circular pile.

**Figure 5:**
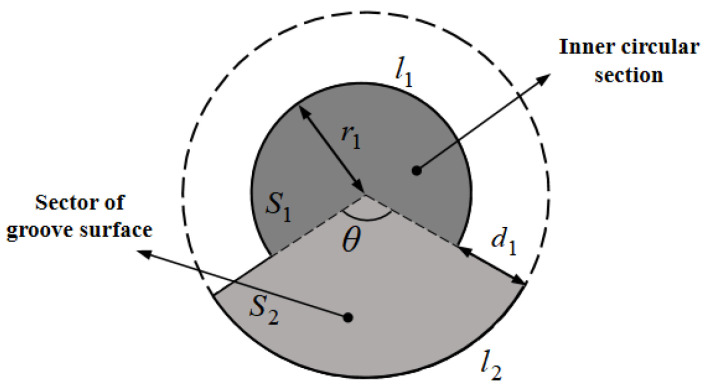
Schematic cross-section of the screw-groove pile.

From the perspective of energy savings, the ratio of the ultimate load of the pile to the volume of the pile is considered the material utilization rate [30]. Nevertheless, the cross-section of the screw-groove pile is complex as shown in Figure 5, and its sectional properties must be analyzed to calculate its volume. The cross-sectional area was calculated by dividing the area into two regions: *S*_1_ and *S*_2_. *S*_1_ is the inner circular section with a radius of *r* and an angle of (2π − *θ*). *S*_2_ is the sector of a grooved surface with a radius of (*r*_1_ + *d*_1_) and an angle of *θ*. Based on the structural analysis of the screw-groove pile shown in Figure 4, the formula for the angle of the region can be expressed as follows:

(1) $$\theta =\frac{2\pi b_{1}}{a_{1}}$$

where *θ* is the angle of the sector of the groove surface (°), *a*_1_ is the screw-groove spacing (m), and *b*_1_ is the thickness of the screw groove (m).

The total cross-sectional area of the pile is denoted as follows:

(2) $$S_{0}=S_{1}+S_{2}=\pi r^{2}\left(\frac{2\pi -\theta }{2\pi }\right)+\pi {\left(r+c_{1}\right)}^{2}\frac{\theta }{2\pi }=r_{1}^{2}\frac{\pi (a_{1}-b_{1})}{a_{1}}+{\left(r_{1}+c_{1}\right)}^{2}\frac{\pi b_{1}}{a_{1}}$$

where *S*_0_ is the cross-sectional area of the screw-groove pile (m^2^), *r*_1_ denotes the inner radius of the screw groove (m), and *c*_1_ denotes the width of the screw groove (m).

The perimeter of the cross-section of the screw-groove pile was calculated based on a combination of three regions: the arc length (*l*_1_) of the inner circular cross-section, the arc length (*l*_2_) of the sector of the groove surface, and the distance (*d*_1_) of the screw groove. Thus, the expression for the perimeter (*l*_0_) of the pile is given by the following:

(3) $$l_{0}=l_{1}+l_{2}+d_{1}=2\pi r_{1}\left(\frac{2\pi -\theta }{2\pi }\right)+2\pi (r_{1}+c_{1})\frac{\theta }{2\pi }+2c_{1}=2\pi (r_{1}+\frac{b_{1}c_{1}}{a_{1}})+2c_{1}$$

where *l*_0_ is the perimeter of the screw-groove pile (m) and *d*_1_ is the distance of the screw groove (m).

The volume of the screw-groove pile can be determined by considering its cross-sectional properties, which account for only 62.93% of the volume of a circular pile with a uniform cross-section. Based on the formula for the material utilization rate of the pile, the screw-groove pile demonstrated a material utilization rate approximately 2.85 times higher than that of the circular pile. This indicates that the screw-groove pile can significantly reduce the amount of material used, thereby achieving energy efficiency, cost reduction, and environmental friendliness. The experimental and numerical analyses of the load–settlement curve trends were highly correlated. Similar to the experimental results, a steep decline was observed when the circular pile reached its ultimate bearing capacity. In contrast, the screw-groove pile displayed a gradual change in behavior without a distinct inflection point. The material utilization rate of the screw-groove pile was 2.94 times higher than that of the circular pile. The difference in the utilization rates between the two falls within 5%, suggesting that the FEM adequately simulated the experimental findings.

### 3.2. Axial Force and Skin Friction

The axial force and skin friction resistance of the circular and screw-groove piles were evaluated as follows:

(4) $$\left\{\begin{array}{l} \sigma =E\times \varepsilon \\ P=\sigma \times A_{c} \end{array}\right.$$

(5) $$q_{s}=\frac{P_{a}-P_{b}}{\pi Dl_{0}}$$

(6) $$p_{n}=E\varepsilon \left(r_{1}^{2}\frac{\pi (a_{1}-b_{1})}{a_{1}}+{\left(r_{1}+c_{1}\right)}^{2}\frac{\pi b_{1}}{a_{1}}\right)$$

(7) $$q_{\mathrm{ss}}=\frac{P_{a}-P_{b}}{2l_{0}\left(\pi (r_{1}+\frac{b_{1}c_{1}}{a_{1}})+c_{1}\right)}$$

where *σ* is the stress of the pile (kPa); *E* is the modulus of elasticity (kPa); *ε* is the strain of the pile; *P* is the axial force of the circular pile (kPa); *A*_c_ is the cross-sectional area of the circular pile (m^2^), *q*_s_ is the skin friction resistance of the circular pile (kPa); *D* is the diameter of the circular pile (m); *P*_a_ and *P*_b_ are the axial forces of the upper and lower sections of the pile (kPa), respectively; *P*_n_ is the axial force of the screw-groove pile (kPa); and *q*_ss_ is the skin friction resistance of the screw-groove pile (kPa).

**Figure 6:**
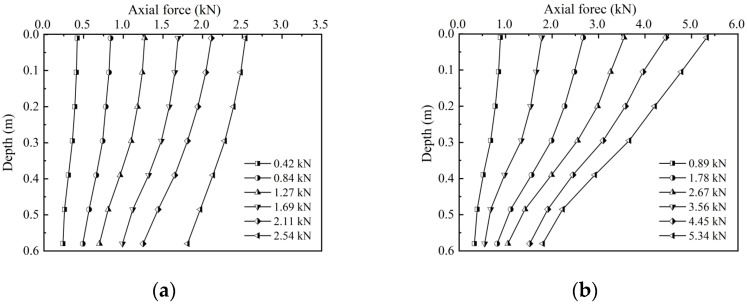
Characteristic curves for axial force distribution of piles: (**a**) circular pile; (**b**) screw-groove pile.

**Figure 7:**
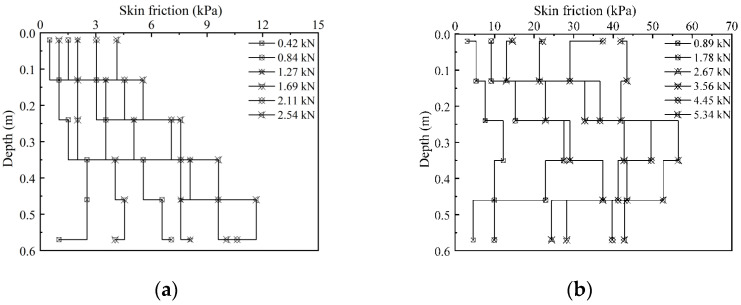
Distribution curves for skin friction resistance of model piles: (**a**) circular pile; (**b**) screw-groove pile.

The characteristic curves of the axial force distributions of the circular and screw-groove piles are shown in Figure 6. The distribution characteristics of the axial force of both pile types decreased gradually along the longitudinal direction of the piles. In contrast, the toe resistance of the pile increased gradually with the load. The decrease in the screw-groove pile was larger than that in the circular pile. Owing to the bearing capacity of the screw-groove structure and the enhancement of the skin friction resistance, the upper load can be shared to reduce the toe resistance of the pile. For the circular pile, when the ultimate load was reached, the toe resistance of the pile was 1.61 kN, approximately 63% of the total ultimate bearing capacity of the pile. Conversely, for the screw-groove pile, with slow settlement deformation, the toe resistance was 4.45 kN and 5.34 kN at loading values of 1.52 kN and 1.81 kN, respectively, which is about 34% of the bearing capacity. Figure 7 illustrates the contrast in the skin friction resistance characteristics between the circular and screw-groove piles. The screw-groove piles exhibited a significantly higher frictional resistance than the circular piles because of their distinctive screw-groove structure, which facilitates interlocking effects with the soil. This enhanced the skin friction between the pile and the soil, increasing the pile-bearing performance. In contrast, the frictional resistance contributed by the cross-sectional area of the circular piles was minimal. In practice, the main difference between circular and screw-groove piles lies in the extent to which the skin frictional resistance is mobilized. At the ultimate load capacity, the frictional resistance of circular piles is primarily derived from the shear strength at the pile–soil interface. In contrast, screw-groove piles benefit from the mechanical interlocking effect of their screw-groove structure. The skin resistance of screw-groove piles encompasses not only the shear strength at the screw-groove surface (comprising the arc length of the inner circular section and the arc length of the groove surface sector) but also a series of base resistances formed by the screw-groove surfaces, thereby cumulatively enhancing the skin resistance of the pile.

### 3.3. Load-Transfer Mechanism

**Figure 8:**
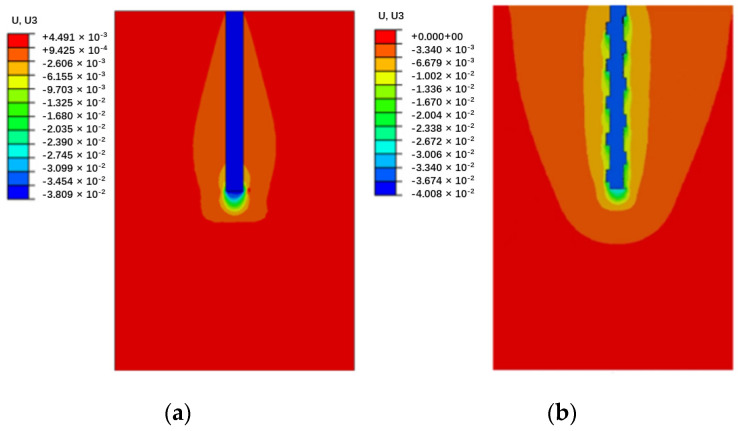
Comparison of pile-soil displacement: (**a**) circular pile; (**b**) screw-groove pile.

**Figure 9:**
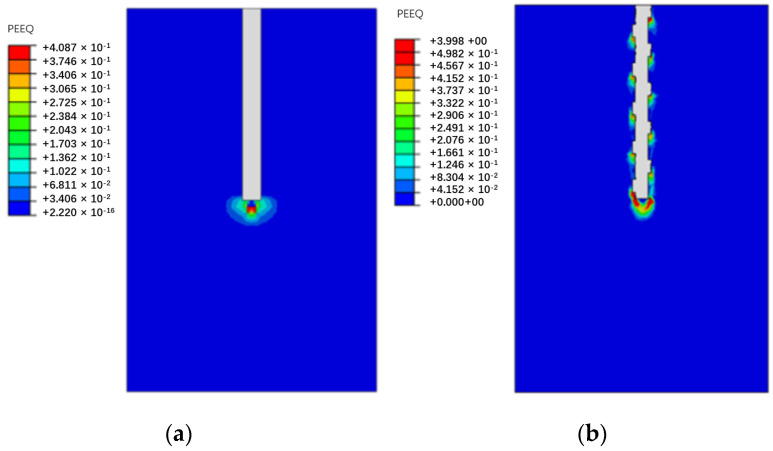
Comparison of plastic deformation for soil: (**a**) circular pile; (**b**) screw-groove pile.

To investigate the load-transfer characteristics of the circular and screw-groove piles in depth, a numerical model was segmented along the X-Z plane as the cutting plane. Figure 7 and Figure 8 illustrate the pile–soil displacement and foundation response characteristics of the piles under ultimate bearing loading conditions. The macroscopic response of the circular pile in the soil involved an evident soil deformation at the pile tip (Figure 8a) and the formation of a high-plasticity strain zone (Figure 9a). Skin resistance arises from the friction between the circular pile and the soil, with the soil layer at the pile tip being the primary load-bearing zone for the pile. In the ultimate bearing state, the vertical displacement range of the screw-groove pile increased significantly, mainly because of the extensive involvement of soil in the bearing capacity through large-scale interaction between the screw-groove structure and the soil. Significant soil deformations (Figure 8b) and highly plastic deformations (Figure 9b) occurred in both the screw-groove structure and the pile tip. The toe resistance at the screw-groove surface originates from the shear strength between the screw-groove structure and the soil around the pile. Therefore, the screw design of the pile increased the contact area between the pile and the soil, thereby enhancing the bearing capacity of the pile. The foundations of the two types of piles exhibited significant differences in their stress characteristics and soil failure modes under vertical loading compared with conventional single-pile end bearing. Unlike ordinary circular piles that rely on single-pile end bearing, screw-groove piles utilize a unique screw-groove structure to form a multipoint end-bearing mode between the screw groove and pile ends. Their unique cross-sectional properties simultaneously enhance both the end-bearing capacity and the skin friction resistance, thereby significantly improving the vertical load-carrying performance of the pile.

**Figure 10:**
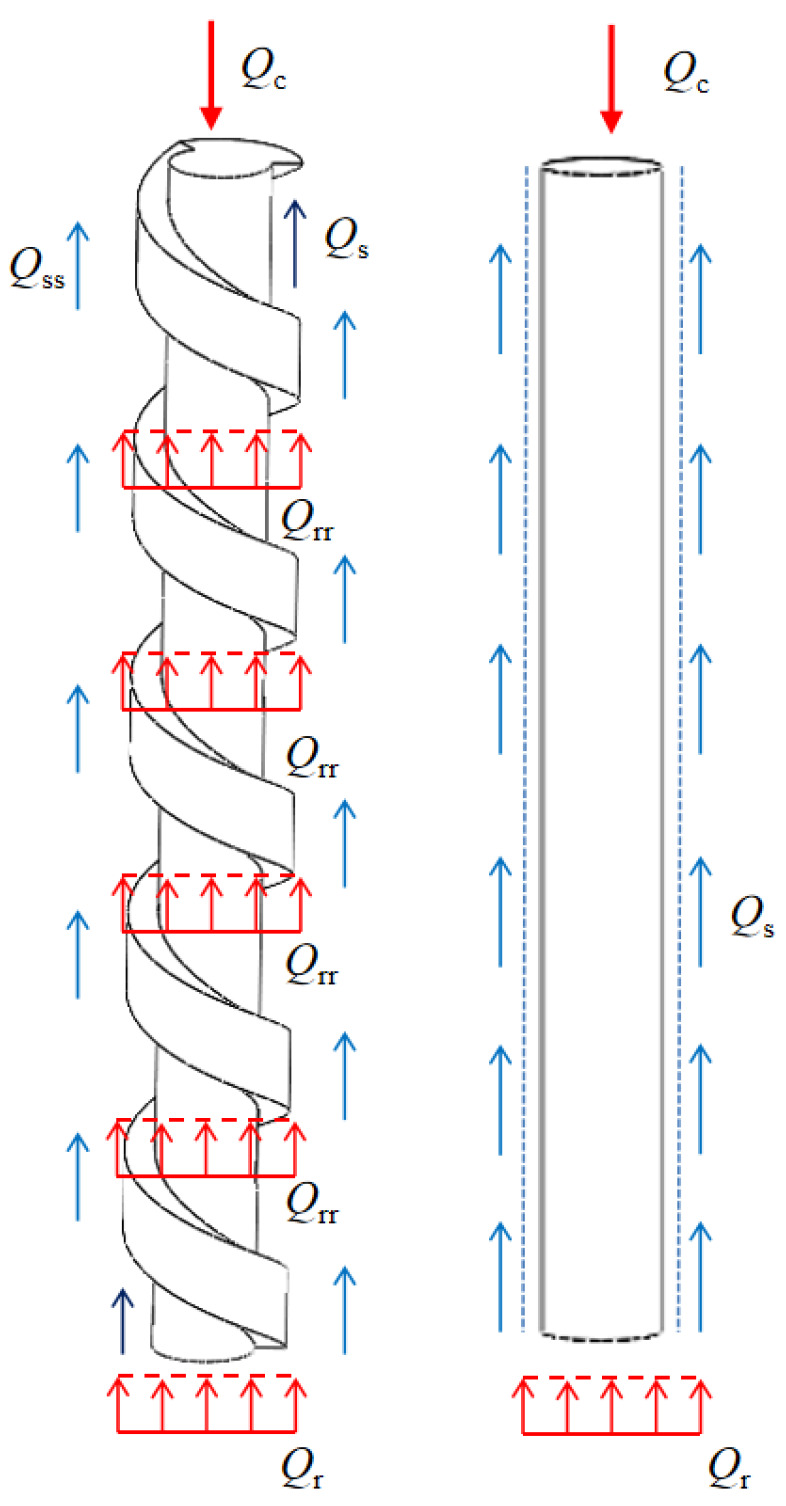
Schematic diagram of load-transfer analysis for screw-groove pile and circular pile.

**Figure 11:**
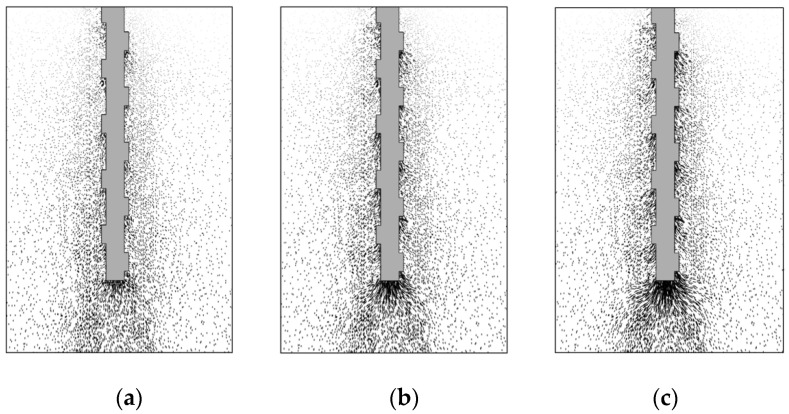
Distribution of stress field of screw-groove pile under different loads: (**a**) 1.78 kN, (**b**) 3.56 kN, and (**c**) 4.71 kN.

A schematic illustrating the load-transfer characteristics of the screw-groove pile based on numerical calculations and experimental results is shown in Figure 10, where the upper part of the pile receives a load and the circular pile is only borne by the pile’s skin friction resistance (*Q*_s_) and toe resistance (*Q*_r_). The load on the screw-groove pile is not only transferred to the screw groove’s inner-diameter skin resistance (*Q*_s_) and toe resistance (*Q*_rr_), but also through the screw groove’s structure. It is transformed by the skin friction resistance of the screw groove (*Q*_ss_) and the screw groove’s bottom-surface resistance (*Q*_rr_) to carry the load jointly. The resistance of the screw groove is similar to that of the pile tip. As shown in Figure 11, the stress distribution at both the screw groove surface end and the pile end gradually increased with increasing load. This aligns with the significant axial force decay observed in the experiments and the high plastic deformation trend at the screw groove surface end observed in the numerical simulations.

### 3.4. Parameter Analysis

#### 3.4.1. Inner Radius of the Screw Groove

**Figure 12:**
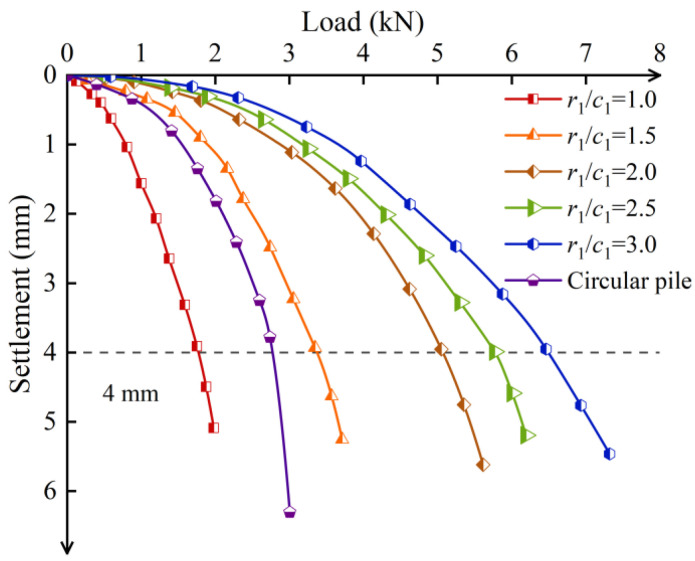
Load–settlement curves of screw-groove piles with different screw-groove inner radii.

Based on the validated FEM, an analysis was conducted to evaluate the effect of the ratio of the inner radius to the width of the screw groove on the bearing capacity of the pile bodies while maintaining a constant groove width and other parameters. The load–settlement curves for different inner radius of the screw groove piles are illustrated in Figure 12. At the initial loading stage, the vertical displacement of the pile body increased linearly with loading. With an increase in load, the displacement of the screw-groove piles changed slowly. Under the same load, the settlement decreased significantly as the inner radius of the screw groove increased. When *r*_1_/*c*_1_ increased from 1 to 3, the ultimate bearing capacity increased by 233.32%. Increasing the inner radius of the pile enhanced the resistance at both the pile–soil interface and the pile tip, thereby contributing to the improvement of the pile’s bearing capacity. It is worth noting, however, that as *r*_1_/*c*_1_ increased from 1.5–2.5 to 2–3, the corresponding increase in the ultimate bearing capacity decreased from 52.4% to 11.4%. This can be attributed to the fact that when the width of the screw groove remains constant and only the radius is increased, the overall incremental effect of the screw groove on the cross-sectional area is relatively minor, as illustrated in Figure 5. Consequently, the incremental influence of the screw-groove structure on the pile’s bearing capacity diminishes, leading to a reduced rate of increase in the ultimate bearing capacity.

**Figure 13:**
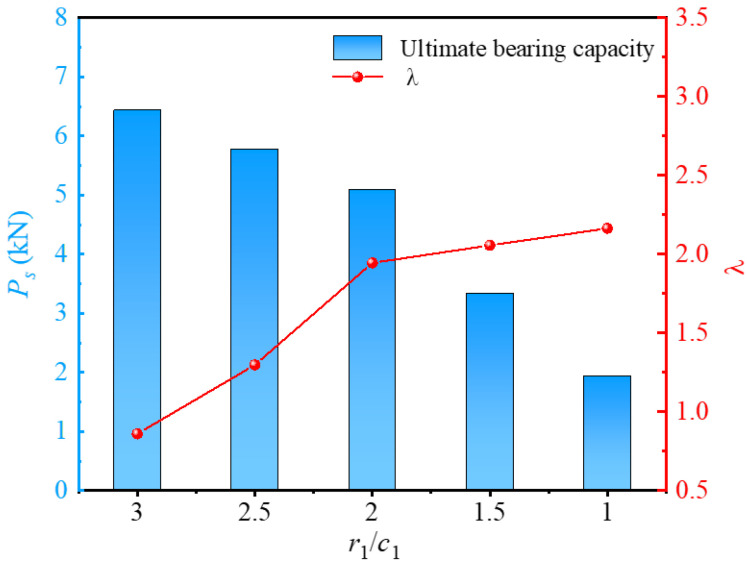
Relationship between ultimate bearing capacity and *λ* for inner radius of screw groove.

Solely analyzing the ultimate bearing capacity reveals that increasing the inner radius leads to a notable enhancement in the bearing capacity. However, this increase in inner radius inevitably increases material consumption, potentially escalating costs and reducing resource utilization efficiency. From an economic and low-carbon perspective, material utilization efficiency is also a crucial indicator in the design and use of precast piles. Based on this, the material utilization factor *λ* = (*L*_b_ − *L*_a_)/*L*, where *L*_a_ is the material utilization rate of piles with circular piles and *L*_b_ is the material utilization rate of screw-groove piles, was also investigated. Figure 13 shows the variation curves of the ultimate bearing capacity (*P_s_*) and material utilization factor under different screw-groove inner radii. However, the ultimate bearing capacity increased with an increase in the inner radius of the screw groove. The utilization factor decreased significantly. As *r*_1_/*c*_1_ increased from 1 to 3, the factor influencing material utilization decreased from 2.16 to 0.86. Decreasing *r*_1_/*c*_1_ from 3 to 1 led to a 151.16% decrease in the utilization factor. Under the conditions of this study, when *r*_1_/*c*_1_ was less than 2, the ultimate bearing capacity of the pile increased significantly. This is because the skin and toe resistances of the pile were significantly enhanced, owing to the influence of the inner radius, and the contact area of the pile–soil interface was significantly increased, thereby strengthening the load-bearing capacity of the pile. However, when *r*_1_/*c*_1_ was greater than 2, although the effect of increasing the pile–soil interface area enhanced the bearing capacity, the material utilization rate decreased significantly. In actual engineering design, the material utilization of the pile should be fully considered while emphasizing the vertical bearing capacity. A huge screw-groove inner diameter can lead to decreased material utilization, whereas a minimal inner diameter results in a poorer load-bearing capacity.

#### 3.4.2. Screw-Groove Spacings

**Figure 14:**
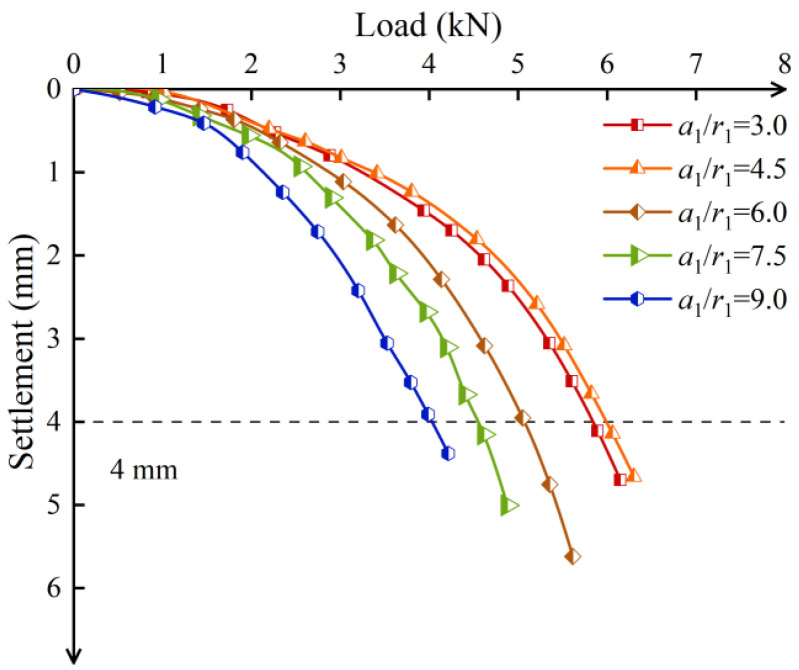
Load–settlement curves of screw-groove piles with different screw-groove spacings.

Figure 14 shows the load–settlement curves of screw-groove piles with different screw-groove spacings. The variation trend of the bearing capacity of the piles with increasing screw-groove spacing generally exhibited a two-stage development characteristic, initially increasing and then decreasing. The ultimate bearing capacities of the piles with spacing-to-inner radius ratios (*a*_1_/*r*_1_) of 3, 4.5, 6, 7.5, and 9 were 5.66 kN, 5.98 kN, 5.09 kN, 4.53 kN, and 4.02 kN, respectively. The screw-groove spacing significantly affected the bearing performance of the pile. When the ratio *a*_1_/*r*_1_ increased from 3 to 4.5, the ultimate bearing capacity increased by 5.65%. However, when *a*_1_/*r*_1_ increased from 4.5 to 9, the ultimate bearing capacity decreased by 32.80%. Minimal spacing can inhibit the transmission effect of the screw-groove pile’s bearing capacity, affecting the diffusion of the pile top load into the surrounding soil and leading to poor bearing performance of the pile. However, a vast pitch reduces the skin friction resistance of the pile and the end-bearing capacity of the screw-groove surface, which is unfavorable for load transfer, thereby weakening the pile–soil shear interaction and resulting in reduced bearing performance.

**Figure 15:**
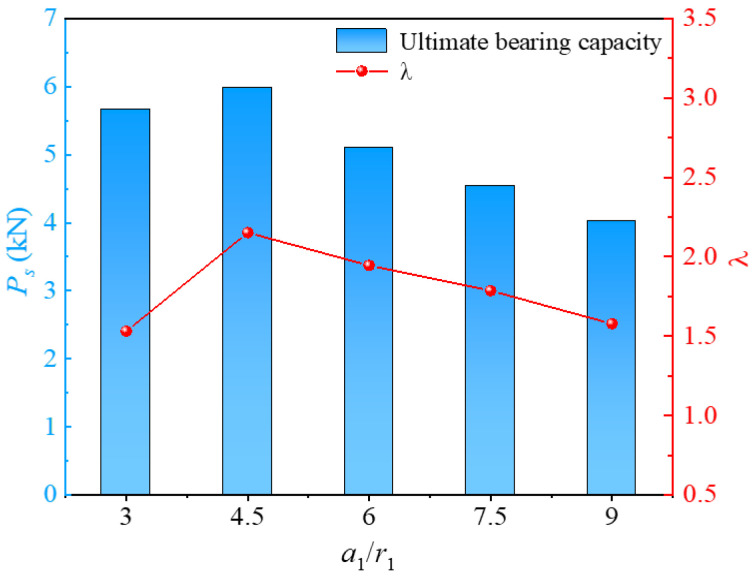
Relationship between ultimate bearing capacity and *λ* for screw-groove spacing.

Figure 15 shows the impact factors of material utilization with increased screw-groove spacing. The material utilization factor of the screw-groove pile and the ultimate bearing capacity exhibited a trend of first increasing and then decreasing. When *a*_1_/*r*_1_ increased from 3 to 4.5, the utilization factor decreased by 40.52%. Conversely, when *a*_1_/*r*_1_ decreased from 9 to 4.5, the utilization factor increased by 36.08%. The trends of changes in the bearing capacity and the utilization rate indicate the existence of an optimal screw-groove spacing that balances economic factors and bearing-capacity demands. Based on the parameters calculated in this study, the optimal ratio was determined to be between 4.5 and 6. In this range, the screw-groove pile effectively mobilizes a larger volume of soil to participate in bearing loads, thereby fully utilizing the bearing capacity of the soil. Consequently, actual engineering designs should consider this optimal screw-groove spacing to meet the dual economic and bearing-capacity needs.

#### 3.4.3. Thickness of Screw Grooves

**Figure 16:**
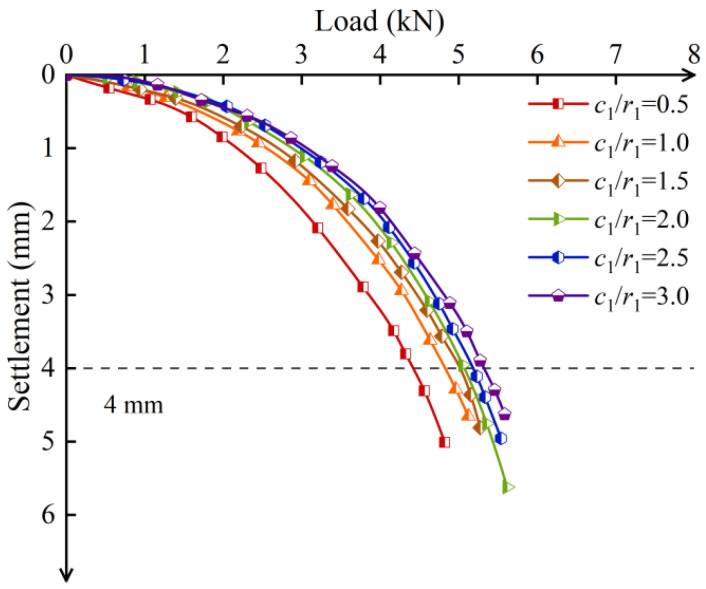
Load–settlement curves of screw-groove piles with different screw-groove thicknesses.

The load–settlement curves for different screw-groove thicknesses are shown in Figure 16. The ultimate bearing capacities for *c*_1_/*r*_1_ at 0.5, 1, 1.5, 2, 2.5, and 3 were 4.43 kN, 4.87 kN, 5.03 kN, 5.09 kN, 5.17 kN, and 5.32 kN, respectively. When *c*_1_/*r*_1_ increased from 0.5 to 1.5, the ultimate bearing capacity increased by 13.52%. However, *c*_1_*/r*_1_ from 1.5 to 3 only resulted in a 5.79% increase. This indicates that increasing the screw-groove thickness can enhance the ultimate bearing capacity, but the rate of increase is limited. Analyzing the increase in the thickness from the perspective of the screw-groove pile cross-section, it only enhanced the pile–soil contact area, thereby increasing the skin friction resistance, with no significant increase in the bearing capacity of the screw-groove structure’s bottom. Therefore, the increase in the load-bearing capacity is limited.

**Figure 17:**
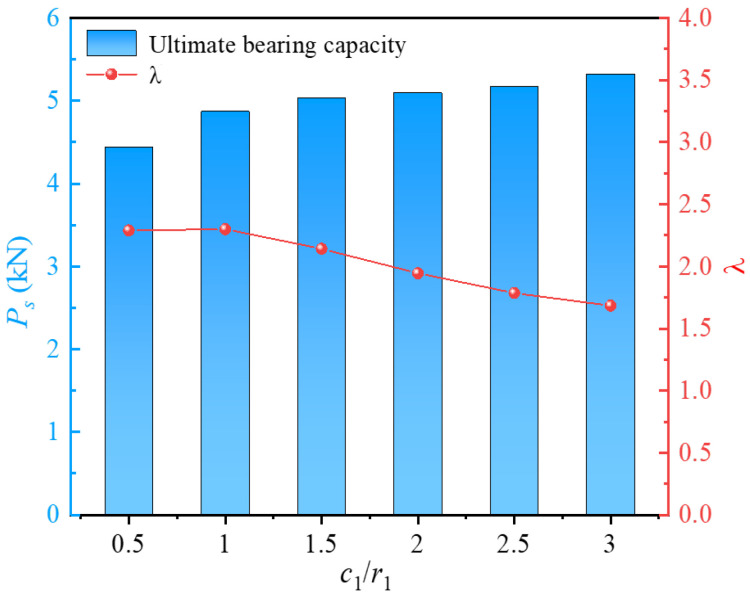
Relationship between ultimate bearing capacity and *λ* for different screw-groove thicknesses.

Figure 17 illustrates the impact factors of material utilization for the *c*_1_/*r*_1_ ratios of 1.5 and 3, which were 2.14 and 1.68, respectively, showing a reduction of 21.50%. The results indicate that when the ratio *c*_1_/*r*_1_ was greater than 1.5, the material utilization of the pile decreased significantly, suggesting that an excessive groove width negatively affects the economic efficiency of practical engineering. When the ratio *c*_1_/*r*_1_ was between 0.5 and 1, the material utilization rates of the screw-groove piles were similar. However, for precast piles with a groove structure, a concrete thickness that is too thin may cause damage at the junction between the groove face end and the inner circular section, compromising the long-term bearing performance of the pile. Therefore, under the experimental conditions of this study, a ratio between 1 and 1.5 is recommended. This range allows the thickness of the screw-groove design to better balance bearing capacity and material utilization.

#### 3.4.4. Soil Parameters

Numerical analysis of soil often employs the Mohr–Coulomb yield criterion, which assumes that the maximum shear stress determines failure. The expression for this criterion is given by

(8) $$\tau_{n}=c+\sigma_{n}\tan\varphi$$

where *τ_n_* is the shear stress (kPa), *c* is the cohesion (kPa), *σ_n_* is the normal stress, and *φ* is the internal friction angle (°). The cohesion and internal friction angle are crucial strength parameters for soils in the model.

**Figure 18:**
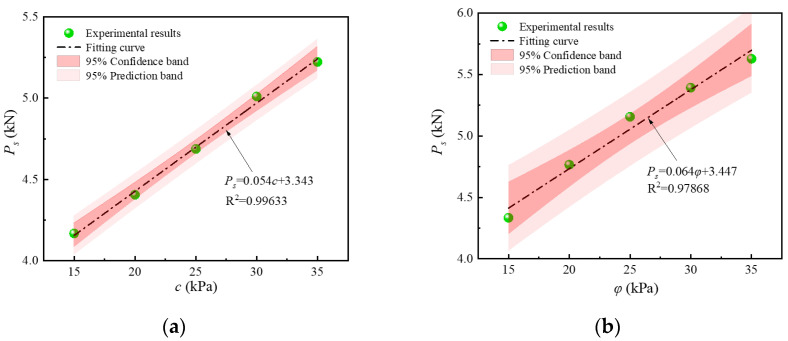
Distribution of ultimate bearing capacity for screw-groove piles: (**a**) cohesion; (**b**) angle of internal friction.

Experimental and analytical calculations showed that the end-bearing capacity of screw-groove piles is strongly correlated with these soil parameters. The Anderson–Darling test [31] was employed to assess the normality of the data, with a significance level set at 0.05. If the *p*-value derived from the test exceeds 0.05, the null hypothesis that the cohesion and internal friction angle follow a normal distribution is accepted. The *p*-values for the two datasets were 0.86 and 0.83, respectively, both exceeding the threshold of 0.05, thereby supporting the assumption that the data followed a normal distribution. Additionally, no outliers [32] or extreme values [33] were detected in the data. Figure 18 illustrates the correlation analysis of different cohesion values and internal friction angles with bearing capacity, revealing correlation coefficients of approximately 0.996 and 0.979, respectively, indicating a good fit. When the cohesion increased from 15.0 kPa to 35.0 kPa, the bearing capacity rose from 4.13 kN to 5.22 kN—an increase of 26.50%. Similarly, when the internal friction angle increased from 15° to 35°, the bearing capacity rose from 4.33 kN to 5.63 kN, representing an increase of 29.86%. The bearing capacity increased with both the cohesion and the internal friction angle. These results indicate that the cohesion and internal friction angle significantly influence the bearing performance of screw-groove piles. The soil parameters affect the shear-bearing capacity of the soil, and the shear strength influences the interlocking action between the screw grooves and the soil. Therefore, the performance of screw-groove piles is highly sensitive to soil parameters, and practical engineering designs must consider the local soil conditions to leverage the inherent bearing capacity of the soil efficiently.

## 4. Conclusions

In this study, the skin friction resistance, axial force, settlement, bearing capacity, and response characteristics of a foundation during vertical bearing of the screw-groove pile and the circular pile were compared and analyzed by combining a model test and FEM. The effects of various parameters (i.e., screw-groove geometric parameters, cohesion, and internal friction angle) on the bearing performance of screw-groove piles were analyzed. Based on the experimental and analytical results, the following conclusions were drawn.

(1) The ultimate bearing capacity of the screw-groove pile was 1.85 times that of the circular pile, with a volume of only 62.93% that of the circular pile, and the material utilization rate was 2.85 times that of the circular pile. Improvements in the material utilization rate can effectively reduce the actual concrete consumption of precast piles in engineering projects, thereby achieving resource conservation goals and a low-carbon economy.

(2) The protruding structure of the screw-groove section significantly enhanced the skin friction resistance of the pile, whereas the shared load bearing at the screw-groove end and pile end formed a multipoint end-bearing load-carrying mode. The bearing performance was strengthened in the end-bearing and friction-resistance aspects compared to piles with a uniform section. Compared with circular piles, the load-bearing range of the soil around the screw-groove pile was significantly increased, and the screw-groove structure enabled more soil to share the load, thereby enhancing the interaction between the soil and the pile. This utilization of the soil-bearing capacity was fully realized.

(3) When the ratio *r*_1_/*c*_1_ increased from 1 to 3, the ultimate bearing capacity increased by 233.32%, whereas the material utilization factor decreased by 151.16%. When *r*_1_/*c*_1_ exceeded 2, the ability to enhance the bearing capacity of the pile through increased pile–soil contact was diminished, significantly reducing the material utilization efficiency. The design of the inner radius of the screw groove for actual engineering needs to consider the balance between bearing capacity and material utilization fully. The thickness of the screw groove had a limited effect on enhancing the ultimate bearing capacity. Under the parameter calculations in this study, when the ratio *a*_1_/*r*_1_ was less than 4.5, the load-transfer effect of the screw-groove pile was inhibited, thereby affecting the diffusion of the pile top load to the surrounding soil. When *a*_1_/*r*_1_ exceeded 6, the end-bearing capacity of the screw-groove surface was reduced, thereby weakening the pile–soil shear interaction and leading to a decrease in the bearing performance. Both the cohesion and internal friction angles significantly affect the bearing performance of the pile. The pile design should be tailored to local soil conditions in practical engineering.

Combined with the research results on the bearing characteristics and material utilization of screw-groove piles, future research will focus on the relationship between bearing efficiency and fabrication costs, considering economic, technological, and environmental factors to optimize the design of screw-groove piles.

## Figures and Tables

**Table 1:** Model material parameters.

	Modulus of Elasticity [MPa]	Density [g/cm^3^]	Poisson Ratio	Cohesion [kPa]	Friction Angle [°]
Pile	7.2 × 10^4^	2.70	0.33	-	-
Soil	8.0	1.81	0.30	33.3	23.4

## Data Availability

The original contributions presented in this study are included in the article. Further inquiries can be directed to the corresponding author.

## Nomenclature

*θ*	Angle of sector of groove surface (°)
*a*_1_	Screw-groove spacing (m)
*b*_1_	Thickness of screw groove (m)
*r*_1_	Inner radius of screw groove (m)
*c*_1_	Width of screw groove (m)
*d*_1_	Distance of screw groove (m)
*S*_0_	Cross-sectional area of screw-groove pile (m^2^)
*l*_1_	Arc length of inner circular cross-section (rad)
*l*_2_	Arc length of sector of groove surface (rad)
*l*_0_	Perimeter of screw-groove pile (m)
*σ*	Stress (kPa)
*E*	Modulus of elasticity (kPa)
*ε*	Strain (dimensionless)
*P*	Axial force (kPa)
*A*_c_	Cross-sectional area of circular pile (m^2^)
*q*_s_	Skin friction resistance of circular pile (kPa)
*D*	Diameter of circular pile (m)
*q*_ss_	Skin friction resistance of screw-groove pile (kPa)
*λ*	Material utilization factor
*L*_a_	Material utilization rate of circular piles
*L*_b_	Material utilization rate of screw-groove piles
*P_s_*	Ultimate bearing capacity (kN)
*τ_n_*	Shear stress (kPa)
*c*	Cohesion (kPa)
*σ_n_*	Normal stress (kPa)
*φ*	Internal friction angle (°)
**List of abbreviations:**	
FEM	Finite element method
